# The components of biodiversity, with a particular focus on phylogenetic information

**DOI:** 10.1002/ece3.3199

**Published:** 2017-07-10

**Authors:** Samuel M. Scheiner, Evsey Kosman, Steven J. Presley, Michael R. Willig

**Affiliations:** ^1^ Division of Environmental Biology National Science Foundation Arlington VA USA; ^2^ Institute for Cereal Crops Improvement Tel Aviv University Tel Aviv Israel; ^3^ Center for Environmental Sciences and Engineering University of Connecticut Storrs CT USA; ^4^ Department of Ecology and Evolutionary Biology University of Connecticut Storrs CT USA

**Keywords:** alpha diversity, bats, beta diversity, gamma diversity, Peru, phylogenetic diversity

## Abstract

We present a framework for biodiversity metrics that organizes the growing panoply of metrics. Our framework distinguishes metrics based on the type of information–abundance, phylogeny, function–and two common properties–magnitude and variability. Our new metrics of phylogenetic diversity are based on a partition of the total branch lengths of a cladogram into the proportional share of each species, including: a measure of divergence which standardizes the amount of evolutionary divergence by species richness and time depth of the cladogram; a measure of regularity which is maximal when the tree is perfectly symmetrical so that all species have the same proportional branch lengths; a measure that combines information on the magnitude and variability of abundance with phylogenetic variability, and a measure of phylogenetically weighted effective mean abundance; and indicate how those metrics can be decomposed into α and β components. We illustrate the utility of these new metrics using empirical data on the bat fauna of Manu, Peru. Divergence was greatest in lowland rainforest and at the transition between cloud and elfin forests, and least in upper elfin forests and in cloud forests. In contrast, regularity was greatest in lowland rainforest, dipping to its smallest values in mid‐elevation cloud forests, and then increasing in high elevation elfin forests. These patterns indicate that the first species to drop out with increasing elevation are ones that are closely related to other species in the metacommunity. Measures of the effective number of phylogenetically independent or distinct species decreased very rapidly with elevation, and β‐diversity was larger. In contrast, a comparison of feeding guilds shows a different effect of phylogenetic patterning. Along the elevational gradient, each guild generally loses some species from each clade–rather than entire clades–explaining the maintenance of functional diversity as phylogenetic diversity decreases.

## 1 INTRODUCTION

The past decade has seen a resurgence in the production of biodiversity metrics. Previous efforts focused primarily on species identity or abundance information (Magurran & McGill, 2011), whereas the current spate has focused on the use of phylogenetic (e.g., Tucker et al., 2017) or functional (e.g., Reynolds, Granados, Lee, & Schroer, 2015) information. The result is a surfeit of metrics but limited clarity about the relative virtues of alternative metrics or how they relate to each other, which can stymy comparative analyzes and complicate attempts at data or conceptual integration. Importantly, it can lead to a mismatch between biodiversity concepts and the metrics designed to measure them.

Biodiversity measures serve two general purposes: understanding the ecological and evolutionary processes that are responsible for or the result of patterns of biodiversity, and directing conservation and management practice that seeks to preserve and exploit biodiversity. To be useful for understanding processes, a biodiversity measure needs to match the process under exploration. For example, if the hypothesized process is competitive exclusion that acts through individual interactions in a local community, then a metric that includes per capita abundance would facilitate the detection of effects of that process. On the other hand, if the hypothesized process is evolutionary divergence acting at a continental extent over millennia, then the metric should not include local per capita values, but should encompass deep evolutionary relationships. To be useful for conservation and management, metrics need common and standardized units. Conservation practice often involves making choices of which location(s) to preserve when it is not possible to preserve all of them (e.g., Bedward, Pressey, & Keith, 1992; Kukkala & Moilanen, 2013). Historically such decisions were predicated on one type of information, species' identities (i.e., species richness). More recent decisions have included information on the phylogenetic relationships or functional roles of those species (e.g., Faith, 1992; Rodrigues & Gaston, 2002; Winter, Devictor, & Schweiger, 2013). Doing so requires that any metrics of taxonomic, phylogenetic, or functional diversity be comparable for locations that contain very different sets of species. For example, metrics that scale with species richness can be effective in putting all information onto a common scale and in communicating conservation outcomes. Metrics that can combine different types of information are desirable when tradeoffs exist between conservation and management goals, such as preserving the maximal amount of evolutionary history, while simultaneously maintaining ecosystem function.

We explore these consideration with respect to three goals. First, we present a general conceptual framework for organizing biodiversity metrics. That framework builds on the work of Mason, Mouillot, Lee, and Wilson (2005) and Villéger, Mason, and Mouillot (2008) for functional trait metrics, of Tucker et al. (2017) for phylogenetic metrics, and Pavoine and Bonsall (2011) for both. Our framework reveals how the types of information typically used to measure biodiversity–abundance, phylogeny, function–are based on two properties–magnitude and variability–thereby clarifying relationships among metrics. The framework makes it easier to discern the attributes that are reflected in composite metrics that include multiple properties of the same or different types of information. In addition, we regularize terms for various properties of biodiversity metrics to facilitate communication and ultimately synthesis.

Building on our recent work on functional diversity (Scheiner, Kosman, Presley, & Willig, 2017), our second goal is to present new metrics for phylogenetic diversity within that framework, including those that combine phylogenetic and abundance information. Although we are adding to the burgeoning number of phylogenetic metrics, our metrics were developed specifically to fill some of the gaps identified by Tucker et al. (2017). Importantly, such variety of phylogenetic and functional metrics is necessary, as one metric cannot effectively capture all aspects of the complex structures of phylogenetic or functional information.

Our third goal is to illustrate the utility of our new metrics based on empirical data of the rich and well‐studied bat fauna from a tropical hot spot (Cisneros et al., 2014; Lewontin, 1995; Patterson, Stotz, Solari, Fitzpatrick, & Pacheco, 1998). Our analyzes of phylogenetic diversity show how such analyzes can complement those of functional diversity for the same ecological system (Scheiner et al., 2017), and provide additional insights into the processes that structure bat communities along elevational gradients in the tropics.

## 2 CREATING A TAXONOMY FOR BIODIVERSITY METRICS

### 2.1 Information types and properties

The core of our general framework for biodiversity metrics recognizes four basic types of information, each with particular properties. Those properties can be combined in a variety of ways to produce many different kinds of metrics. Moreover, those basic properties may be quantified in more than one fashion. In this study, we are agnostic about the relative virtues of how those properties are measured or combined. Our purpose is to comprehensively organize the metrics as a first step for enhancing comparative or synthetic understanding. For ease of presentation, we couch this discussion with respect to species, but emphasize that our framework can be used for entities such as genes, individuals, or communities. Biodiversity is typically measured for a set of species, which we represent as communities within a landscape, although that set could also consist of an ensemble, a clade, or other focal entity.

The four basic types of information about each species are as follows: identity, abundance, phylogeny, and function. With regard to measuring biodiversity, the four types of information differ in that identity and absolute abundance are aspects of a species that are not mathematically dependent on any other species in the set. In contrast, phylogenetic and functional diversities are aspects that can only be measured relative to other species. Three of those types of information–abundance, phylogeny, function–evince two properties: magnitude and variability (Table 1). We chose this terminology for these properties because it is generic enough to encompass the many different metrics that have been proposed.

**Table 1:** A proposed nomenclature for and examples of properties of the three types of information

Information type	Magnitude property	Example	Variability property	Example
Abundance	Numbers	McIntosh's U index (McIntosh, 1967)	Evenness	Simpson index (Simpson, 1949)
Phylogeny	Divergence	Mean branch length (avPD) (Clarke & Warwick, 2001)	Regularity	Branching symmetry (*I*_*C*_) (Colless, 1982)
Function	Dispersion	Mean pairwise distance (M) (Scheiner et al., 2017)	Equability	Functional‐trait evenness [^*q*^ *E*(*T*)] (Scheiner et al., 2017)

Magnitude quantifies how much each of the species in the set manifests some property. For abundance, magnitude is typically the total number of individuals of a particular species, although it can be measured in a variety of other ways such as frequency of occurrence, biomass, or geographic range; for simplicity in this study, we focus on numbers of individuals. For phylogeny, magnitude is the amount of evolutionary differentiation of a particular species from other species, which Tucker et al. (2017) term “divergence.” For function, magnitude is the difference in trait values of a particular species from all other species, which Mason et al. (2005) term “functional richness,” Carmona, de Bello, Mason, and Lepš (2016) term “functional distinctiveness” and Scheiner et al. (2017) term “dispersion.” Pavoine and Bonsall (2011) use the term “divergence” to refer to the magnitude property of both phylogenetic and functional information. We propose the use of different terms for the two types of information to minimize confusion as to which type of information is being quantified.

Variability quantifies the extent to which magnitudes differ among those species. For abundance, variability is the evenness of the (relative) number of individuals of each species. For phylogeny, variability is the extent to which species are equally divergent, which Tucker et al. (2017) term “regularity.” For function, variability is the extent to which species are equally different from each other in trait values, which we term “equability” and which Villéger et al. (2008) term “functional evenness.” [An alternative set of terms for the variability property–abundance evenness, phylogenetic evenness, functional evenness–would make transparent the similarity of properties among the types of information.] Pavoine and Bonsall (2011) use the term “regularity” to refer to the variability property of both phylogenetic and functional information.

### 2.2 Combining types of information

Many biodiversity metrics are created by combining properties of the same or different types of information. For example, the original Hill diversity [^*q*^ *D*(*A*)] (Hill, 1973) was a combination of abundance evenness and species richness. What Tucker et al. (2017) identify as a third property of phylogeny information–”richness”–is actually a composite of phylogenetic divergence and species richness, which we demonstrate below using Faith's PD (Faith, 1992). Moreover, a particular metric can include more than two properties, as in Scheiner et al. (2017), where a metric of functional diversity combined dispersion, equitability, and species richness. Such composite metrics can be used for examining ecological or evolutionary processes that jointly affect those properties (e.g., ecosystem processes that are affected by the relative abundances of functionally different species) and for putting metrics into a common currency (e.g., the effective number of species). Below, we show how such combinations of properties can be achieved with phylogenetic information.

Families of biodiversity metrics can be defined based on the inclusion of particular properties. For example, metrics based on the Hill formulation combine identity (i.e., species richness) with variability of abundance (Hill, 1973), phylogeny (Chao, Chiu, & Jost, 2010; Scheiner, 2012), or function (Chiu & Chao, 2014; Scheiner et al., 2017). In doing so, each provides a composite metric scaled to species richness (the effective number of species). Many metrics of functional diversity combine abundance information with functional information (e.g., Chiu & Chao, 2014; Laliberté & Legendre, 2010; Scheiner et al., 2017; Villéger et al., 2008). Families of metrics can also be defined by the use of a specific measure of a property, for example, the use of Rao's Q (Rao, 1982) to measure the magnitude of phylogenetic divergence or functional dispersion.

These families need not be mutually exclusive. Total functional diversity (Chiu & Chao, 2014) uses Rao's Q within a Hill formulation and could be considered to be associated with two different families of metrics. Families can, thus, be defined in many different ways based on the types of information or properties included, ways of measuring those properties, or mathematical approaches for computing the metrics. We are not advocating for any particular types, measures, or approaches. Rather, our framework clarifies the ways that metrics are or are not related, which will make assessments or comparisons of metric properties clearer (e.g., Scheiner et al., 2017; Tucker et al., 2017).

For some composite metrics, the combined properties are not always obvious. One strategy for determining those properties is to see how the metric behaves when one part is constant (e.g., all species are equally abundant, divergent, or distinctive) and other parts vary. We provide an example of this approach in our comparison of various metrics of phylogenetic diversity that combine abundance and phylogenetic information (see below).

### 2.3 Terminology and symbols

Along with the proliferation of biodiversity metrics has come a plethora of terms and symbols. Unfortunately, it is only in hindsight, after various terms and notations have become embedded in the literature, that we recognize a need for more precise or explicit verbiage or symbols as a way to reduce confusion or enhance incisiveness (e.g., Tuomisto, 2010a, 2010b). Because abundance‐based metrics and species richness are the oldest representations of biodiversity, they are often used without modifiers. For example, the Hill approach has been applied to both phylogenetic and functional data, but the metric based on abundance data is just called “Hill diversity.” Because it was the first, Faith (1992) simply called his metric “phylogenetic diversity” (PD), even though it is just one of many metrics that measure aspects of phylogenetic diversity.

Rather than try to impose new names on old concepts, usually a quixotic endeavor, we urge that new concepts be given clear and descriptive names. For example, we recommend that the “richness” of Tucker et al. (2017) be called “phylogenetic richness” to avoid confusion with species richness. On the other hand, symbols can be standardized more readily. Thus, Scheiner (2012) proposed that Hill diversity based on abundance data be symbolized as ^*q*^ *D*(*A*), rather than as ^*q*^ *D*, as was previously done so as to distinguish it from Hill diversity using phylogenetic [^*q*^ *D*(*P*)] or functional trait [^*q*^ *D*(*T*)] information. Although it is not possible or necessary to standardize all symbols, we urge cognizance of the need for clarity when proposing new metrics.

## 3 PHYLOGENETIC DIVERSITY METRICS

### 3.1 A composite metric of phylogenetic diversity

We present a new composite metric (Table 2) that builds on that of Scheiner (2012). He presented a metric based on the variability property and species richness, using a Hill (1973) approach. Here, we show how the composite metric is related to a metric based on magnitude (divergence) and indicate how those metrics can be decomposed into α and β components. We also present a new metric that combines phylogenetic and abundance information, and contrast it with the metrics of Chao et al. (2010) and Scheiner (2012).

**Table 2:** The metrics of phylogenetic diversity defined in this study

Name	Symbol	Description	Formula	Properties
Mean proportional divergence	*M*(*P*)	Time‐depth standardized mean branch length	$\left(\sum_{i}^{S}\sum_{\in b\left(S_{i}\right)}L_{ij}\right)/\left(T\times S\right)$	Divergence
Phylogenetic Hill evenness	^*q*^ *E*(*P*)	Symmetry of branch lengths	${\left(\sum_{i}^{S}{\left(\frac{\sum_{\in b\left(S_{i}\right)}L_{ij}}{\sum_{i}^{S}\sum_{\in b\left(S_{i}\right)}L_{ij}}\right)}^{q}\right)}^{1/\left(1-q\right)}/S$	Regularity
Chao's phylogenetic richness	*M*(*PR*)	Effective number of phylogenetically independent species	$\left(\sum_{i}^{S}\sum_{\in b\left(S_{i}\right)}L_{ij}\right)/T$	Identity, divergence
Phylogenetic Hill distinctiveness	^*q*^ *D*(*P*)	Effective number of equally phylogenetically distinct species	${\left(\sum_{i}^{S}{\left(\frac{\sum_{\in b\left(S_{i}\right)}L_{ij}}{\sum_{i}^{S}\sum_{\in b\left(S_{i}\right)}L_{ij}}\right)}^{q}\right)}^{1/\left(1-q\right)}$	Identity, regularity
Abundance‐weighted phylogenetic Hill evenness	^*q*^ *E*_*I*_(*AP*)	Symmetry of branch lengths weighted by abundance	${\left(\sum_{i}^{S}{\left(\frac{\sum_{\in b\left(S_{i}\right)}L_{ij}^{\prime }}{\sum_{i}^{S}\sum_{\in b\left(S_{i}\right)}L_{ij}^{\prime }}\right)}^{q}\right)}^{1/\left(1-q\right)}/S$	Numbers, evenness, divergence
Abundance‐weighted phylogenetic Hill distinctiveness	^*q*^ *D*_*I*_(*AP*)	Effective number of equally abundant and phylogenetically distinct species	${\left(\sum_{i}^{S}{\left(\frac{\sum_{\in b\left(S_{i}\right)}L_{ij}^{\prime }}{\sum_{i}^{S}\sum_{\in b\left(S_{i}\right)}L_{ij}^{\prime }}\right)}^{q}\right)}^{1/\left(1-q\right)}$	Identity, numbers, evenness, divergence
Phylogenetic abundance	*A*(*P*)	Phylogenetically weighted effective mean abundance	$\frac{\sum_{i}^{S}n_{i}}{{\left(\sum_{i}^{S}{\left(\frac{\sum_{\in b\left(S_{i}\right)}L_{ij}^{\prime }}{\sum_{i}^{S}\sum_{\in b\left(S_{i}\right)}L_{ij}^{\prime }}\right)}^{q}\right)}^{1/\left(1-q\right)}}$	Identity, numbers, evenness, divergence

*S*: number of species; *N*: total number of individuals; *n*_*i*_: the number of individuals in the *i*th species; *T*: time depth of the cladogram; *L*_*ij*_: the proportional share of the *j*th branch segment of the *i*th species; $L_{ij}^{\prime }$: the abundance‐weighted proportional share of the *j*th branch segment of the *i*th species.

Our phylogenetic metric is based on partitioning the total branch lengths of a cladogram into the proportional share associated with each species. That partition forms the basis of other phylogenetic diversity metrics such as summed evolutionary distinctiveness (ED) and average phylogenetic diversity (avPD). Tucker et al. (2017, appendix S1) provide formulas and citations for those and related metrics, and Chao et al. (2010) and Scheiner (2012) provide detailed explanations of how to calculate branch length characteristics.

### 3.2 Divergence

We define a metric of divergence in a way that makes clear its relationship to those previously defined. For convenience, our definition assumes an ultrametric tree, that is, all species have equal total lengths from the root to the tip. For example, a time‐calibrated tree of extant species is ultrametric. However, that assumption is not necessary. A common type of nonultrametric tree is one based on the amount of change in DNA. For such trees, time depth (*T*) is replaced by the mean distance from the root to the tips of the tree [see Chao et al. (2010) and Chiu, Jost, and Chao (2014) for discussions of such calculations].

If *L*_*j*_ is the length of the *j*th branch segment of a cladogram of *S* species, *S*_*j*_ is the number of species that share the *j*th branch, and *b*(*S*_*i*_) is the set of branches in the path from the root to the tip of the *i*th species, then *L*_*ij*_ = *L*_*j*_/*S*_*j*_ is the proportional share of the *j*th branch segment of the *i*th species for each branch *j* that belongs to *b*(*S*_*i*_). Each branch that extends from the root of the cladogram represents an independent clade, and the proportional shares assigned to each species are determined within each independent clade. The total branch length of the entire cladogram is as follows:

(1) $$B=\;\sum_{i}^{S}\sum_{j\in b\left(S_{i}\right)}L_{ij}$$

This metric is Faith's PD. From this total, we define a measure of divergence, mean proportional branch length:

(2) M(P)=B/(T×S)

This metric standardizes the amount of evolutionary divergence by species richness (*S*) and time depth (*T*) of the cladogram and has a range of [1/*S*,1]. *M*(*P*) is maximal when all species are phylogenetically independent so that they diverge at the root of the cladogram (i.e., a star phylogeny). For cladograms with the same topology and time depth, one that has longer branches toward the tips will have greater values than a cladogram with longer branches toward the root.

The double standardization (by *S* and *T*) has the advantage of allowing meaningful comparisons among trees that differ in either species richness or time depth, a problem that plagues Faith's PD (see Clarke & Warwick, 2001, figure 6). After rearranging Equation (2) as *B*/*T *= *S *× *M*(*P*), it is obvious that total branch length (Faith's PD) represents a composite metric that combines species richness (identity) with divergence (phylogenetic magnitude). As a result, differences in Faith's PD among datasets often reflect differences in species richness, rather than differences in phylogenetic patterns.

When abundance, phylogenetic, or functional information is combined with identity information, the resulting metric is in units of effective numbers of species. Such units are particularly useful because they are meaningful for management purposes (we typically want to conserve species richness or its proxies) and can be related to ecological or evolutionary processes that are linked to species richness. For divergence, such a metric is given by the time‐standardized mean branch length:

(3) M(PR)=S×M(P)=B/T,

which measures the effective number of phylogenetically independent species. We term it “Chao's phylogenetic richness” (see below) because of its relationship to the phylogenetic diversity metric defined by Chao et al. (2010).

Our measure of divergence is closely related to mean branch length (Clarke & Warwick, 2001), in that avPD = *B*/*S*, which has a range of [*T*/*S*,*T*]. This metric standardizes Faith's PD with respect to species richness, but not time depth. For comparisons among taxa or study systems, avPD might be more appropriate than *M*(*P*) if the absolute amount of divergence is of interest, for example with nonultrametric trees. When a cladogram contains the entire species pool, mean(ED) is equivalent to avPD (Tucker et al., 2017).

### 3.3 Regularity

If *L*_*i*_ = Σ*L*_*ij*_ is the total lineage divergence of the *i*th species, then *l*_*i*_ = *L*_*i*_/*B* is the proportional lineage divergence of the *i*th species. [*L*_*i*_ is the equivalent of ED_*i*_ as defined by Redding (2003) and Isaac, Turvey, Collen, Waterman, and Baillie (2007).] We then use the Hill formulation to define a measure of regularity, phylogenetic Hill evenness:

(4) $${}^{q}E\left(P\right)={\left(\sum_{i}^{S}l_{i}^{q}\right)}^{1/(1-q)}/S.$$

This metric has the range (1/*S*,1]. It is maximal when the tree is perfectly symmetrical so that all species have the same proportional branch lengths (for an example, see figure 2D of Scheiner, 2012). The exponent (*q*) weights the relative contributions of species so that highly divergent species are given disproportionately more weight with greater values of *q*. When *q *=* *0, the formula reduces to species richness. When *q *=* *1 a limit formulation must be used:

(5) $${}^{1}E\left(P\right)=\text{exp}\left(-\sum_{i}^{S}l_{i}\log l_{i}\right)/S.$$

This last metric is the equivalent of *E*_ED_ of Cadotte et al. (2010); our metric is a generalization of that formulation.

From our metric, we derive another composite metric, phylogenetic Hill distinctiveness, ^*q*^ *D*(*P*) = *S *× ^*q*^ *E*(*P*), which is the phylogenetic diversity metric defined by Scheiner (2012). It measures the effective number of equally phylogenetically distinct species in a community and has a range of (1,*S*]. It combines species richness (identity information) with regularity (variability information about phylogeny). When *q *=* *1, it is the equivalent of *H*_ED_ of Cadotte et al. (2010); our metric is a generalization of that formulation.

Hill numbers obey a doubling principle (Chao et al., 2010; Chiu & Chao, 2014). For abundance information, if a set of species with particular abundances is replicated (i.e., the additional set contains the same number of species and the same abundance distribution as the original set, and the two sets share no species), then the Hill diversity will double. For phylogenetic information, replication is the equivalent of adding a second cladogram, identical to the first, joined at the root. In that case, ^*q*^ *D*(*P*) would double. If no species were added, but all branch lengths were doubled, ^*q*^ *D*(*P*) would not change.

### 3.4 Decomposing metrics into α and β components

Often we want to examine biodiversity with respect to whole‐part relationships, such as communities within a metacommunity or landscape, or guilds within a clade. By convention, we refer to the biodiversity of the entire set (e.g., landscape) as the γ component, the mean biodiversity within subgroups (e.g., communities) as the α component, and the variation among subgroups as the β component. Typically, such whole‐part relationships refer to spatial subgroups, but need not. In our bat example, we partition biodiversity in two ways: by spatial subgroups and by foraging guilds.

For each of our proposed metrics, the whole‐part relationship is multiplicative:

(6) $$\begin{array}{cc} & M{\left(P\right)}_{\gamma }\;=\;M{\left(P\right)}_{\alpha }\;\times \;M{\left(P\right)}_{\beta }\\ & M{\left(PR\right)}_{\gamma }\;=\;M{\left(PR\right)}_{\alpha }\;\times \;M{\left(PR\right)}_{\beta }\\ & {}^{q}E{\left(P\right)}_{\gamma }\;={\;}^{q}E{\left(P\right)}_{\alpha }\;\times {\;}^{q}E{\left(P\right)}_{\beta }\\ & {}^{q}D{\left(P\right)}_{\gamma }\;={\;}^{q}D{\left(P\right)}_{\alpha }\;\times {\;}^{q}D{\left(P\right)}_{\beta } \end{array}$$

In all instances, the α component is calculated as the average of the values for each subgroup, and the β component is calculated by dividing the γ component by the α component. Metrics that include identity information–*M*(*PR*)_β_ and ^*q*^ *D*(*P*)_β_–can be interpreted as the effective number of subgroups. For example, if the subgroups are communities in a landscape, ^*q*^ *D*(*P*)_β_ measures the effective number of communities that would contain an equal number of equally divergent species. Our metrics of ^*q*^ *E*(*P*)_β_ and ^*q*^ *D*(*P*)_β_ provide measures of a β component for regularity, for which Tucker et al. (2017) noted a lack of current metrics. When computing the α component, the branch length values for each species must be those from the cladogram of the entire set, not separately from the cladograms of each of the subgroups. For abundance data, disagreement exists about how averages for the α component should be calculated, whether to use weighted or unweighted values of the subgroups (Jost, 2007; Tuomisto, 2010a). We leave that as an unresolved issue, as either approach could be applied within this framework. For our bat example, we computed unweighted averages.

### 3.5 Combining with abundance information

Many biodiversity metrics combine abundance information with phylogenetic or functional information. Here, we treat abundance as the number of individuals of a species, while noting that for taxonomic data other sorts of weightings could be used (e.g., focusing on the biodiversity of genera weighted by either the number of species within each genus or the total number of individuals within each genus regardless of species identity).

To obtain our measure of divergence weighted by abundance, let *n*_*i*_ be the number of individuals in the *i*th species and *N*_*j*_ be the total number of individuals of all species that share the *j*th branch segment. Then $L_{ij}^{\prime }$ = *n*_*i*_ *L*_*j*_/*N*_*j*_ is the proportional share of the *j*th branch segment of the *i*th species weighted by its relative abundance. To obtain our measure of regularity weighted by abundance, let $l_{i}^{\prime }=\sum_{j\in b\left(S_{i}\right)}L_{ij}^{\prime }/B$. In general, $l_{i}^{\prime }$ ≠ *l*_*i*_ as defined previously because shared branches are now weighed by proportional abundance. Our abundance‐weighted measure of regularity is then:

(7) $${}^{q}E_{I}\left(AP\right)={\left(\sum_{i}^{S}{l^{\prime }}_{i}^{q}\right)}^{1/\left(1-q\right)}/S,$$

where *A* indicates that the metric is weighted by abundance. This metric has a range of (1/*S*,1], and the limit formula (Equation (4) with $l_{i}^{\prime }$ instead of *l*_*i*_) should be used when *q* = 1.

As before, we can define a composite metric, ^*q*^ *D*_*I*_(*AP*) = *S *× ^*q*^ *E*_*I*_(*AP*), with a range of (1,*S*] that measures the effective number of equally phylogenetically distinct species weighted by abundances. We use the subscript “I” to indicate that divergence is weighted by the number of individuals and to distinguish this metric from that defined by Scheiner (2012). When all species are equally abundant, ^*q*^ *D*_*I*_(*AP*) = ^*q*^ *D*(*P*). Even when all are not equally abundant, if species within each independent clade are equally abundant, it is also the case that ^*q*^ *D*_*I*_(*AP*) = ^*q*^ *D*(*P*) because members of independent clades share no branches. Thus, ^*q*^ *D*_*I*_(*AP*) is a function of the joint distribution of abundance and divergence. The metric depends on how abundances are distributed across the cladogram, rather than variability alone, so that ^*q*^ *D*_*I*_(*AP*) is affected by both the magnitude and the variability of the abundances. As the most divergent species within a clade become more abundant, ^*q*^ *D*_*I*_(*AP*) becomes larger because species on more distant branches are dominating the community and phylogenetic evenness is increasing. As the least divergent species become more abundant, ^*q*^ *D*_*I*_(*AP*) becomes smaller because species on more central branches are dominating the community, and phylogenetic evenness is decreasing. Moreover, ^*q*^ *D*_*I*_(*AP*) obeys the doubling principle. If the cladogram is replicated at the root and the new half contains the same topology, branch lengths, and abundances, diversity doubles. On the other hand, if all abundances double, diversity is unchanged.

This metric differs from that of Scheiner (2012):

(8) $${}^{q}D\left(AP\right)={\left(\sum_{i}^{s}{\left(\frac{n_{i}L_{i}}{\sum n_{i}L_{i}}\right)}^{q}\right)}^{1/1-q},$$

in which the branch lengths are not weighted by abundance. Consequently, when all species are equally divergent, ^*q*^ *D*(*AP*) = ^*q*^ *D*(*A*), rather than ^*q*^ *D*(*P*). On the other hand, when all species are equally abundant, ^*q*^ *D*(*AP*) = ^*q*^ *D*(*P*), as is the case for ^*q*^ *D*_*I*_(*AP*).

Chao et al. (2010) combined abundance and phylogenetic information using a Hill approach in a different fashion:

(9) $${}^{q}D{\left(P\right)}^{T}=\frac{1}{T}{\left(\sum_{b\in B_{t}}\frac{L_{b}}{T}p_{b}^{q}\right)}^{1/1-q},$$

where *L*_*b*_ is the length of branch *b*, and *p*_*b*_ is the sum of the relative abundances of the species that share that branch. [We notate this metric as ^*q*^ *D*(*P*)^*T*^ rather than ^*q*^ *D*(*T*) as in Chao et al. (2010) to distinguish it from the metric of functional‐trait diversity in Scheiner et al. (2017).] When all species are equally divergent, ^*q*^ *D*(*P*)^*T*^ **= ^*q*^ *D*(*A*), as it reduces to a Hill diversity based on relative abundances. When all species are equally abundant, ^*q*^ *D*(*P*)^*T*^ = *B*/*T *= *M*(*PR*), Chao's phylogenetic richness as defined previously. From this, it is clear that ^*q*^ *D*(*P*)^*T*^ encompasses abundance variability and phylogenetic magnitude. In contrast, ^*q*^ *D*(*AP*) encompasses abundance variability and phylogenetic variability, whereas ^*q*^ *D*_*I*_(*AP*) encompasses abundance magnitude and variability along with phylogenetic variability. Thus, these three approaches for combining abundance and phylogenetic information differ in the properties that they combine.

When we only take species identity into account, the mean abundance of the species in a community is $\bar{N}=\sum_{i}^{S}n_{i}/S$. We can define a related concept, the phylogenetically weighted effective mean abundance:

(10) $$A\left(P\right)=\sum_{i}^{S}n_{i}/{}^{q}D_{I}\left(AP\right)=\bar{N}/{}^{q}E_{I}\left(AP\right).$$

The divisor is the effective number of phylogenetically distinct species, rather than species richness. That divisor now includes information on regularity; that is, mean abundance is now weighted by phylogenetic evenness. Because ^*q*^ *D*_*I*_(*AP*) ≤ *S*,* A*(*P*) ≥ $\overset{¯}{N}$ and the effective mean abundances are minimized when all species are equally abundant and equally divergent.

## 4 AN EXAMPLE WITH PERUVIAN BATS

We illustrate the use of our phylogenetic diversity metrics with data that characterizes the bat fauna of the Manu Biosphere Reserve (hereafter Manu, Patterson, Stotz, & Solari, 2006). This same dataset was used to demonstrate the properties of our metric of functional diversity (Scheiner et al., 2017). Manu is located on the eastern slopes of the Andes in southeastern Peru (Terborgh, 1971). It spans an extensive elevational range (340–3,625 m asl) and supports structurally and compositionally distinct vegetation types that occur sequentially along the elevational gradient (Lewontin, 1995; Presley, Scheiner, & Willig, 2014). Vegetation varies from lowland rainforest (<500 m asl) with 50 to 60 m canopies, to patches of elfin forest (>3,200 m asl) characterized by a low canopy (3–5 m) and dense vegetation intermixed with tall grasslands.

**Figure 1:**
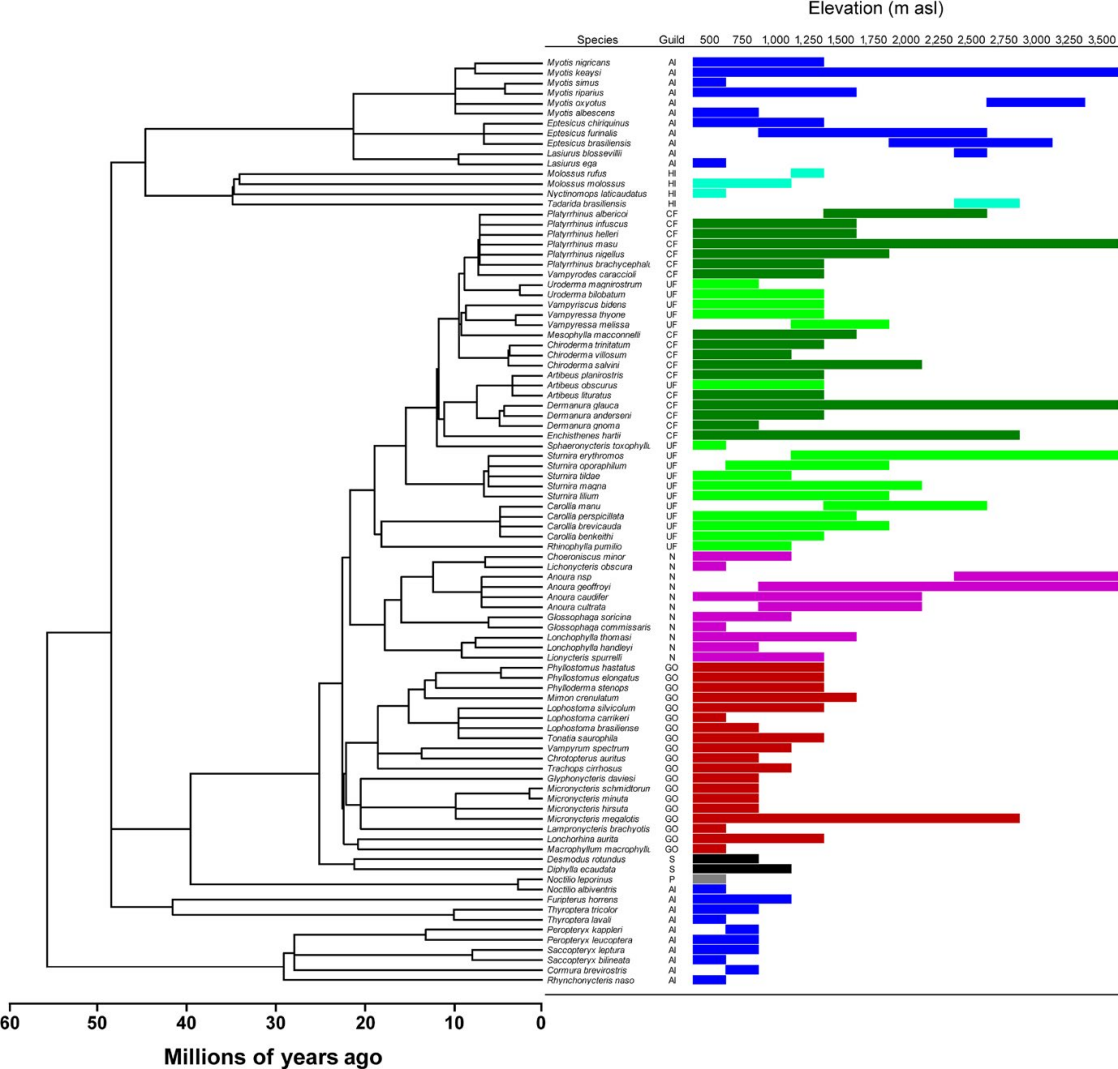
The cladogram for the bats at the Manu Biosphere Reserve with indications of guild membership and elevational distribution for each species. AI, aerial insectivore (dark blue); CF, canopy frugivore (dark green); GO, gleaning omnivore (red); HI, high‐flying insectivore (light blue); N, nectarivore (pink); P, piscivore (gray); S, sanguinivore (black); UF, understory frugivore (light green)

The elevational distributions of the bat species were based on comprehensive surveys conducted over many years (table S2 of Patterson et al., 2006). Data on species incidence were organized into thirteen 250 m elevational strata, with each stratum denoted as a community for our analyzes. The stratum extents were chosen to balance the resolution of empirical records with collection effort (Patterson et al., 1998). Phylogenetic relationships were based on branch lengths from a species‐level supertree for bats (Jones, Bininda‐Emonds, & Gittleman, 2005; Figure 1). Ten of the 92 species from Manu were not present in this supertree, four of which are newly described species. The closest congener present in the supertree that was not from Manu was substituted for each missing species (Cisneros et al., 2014). The effects of these substitutions on calculations of phylogenetic diversity likely were small because the lengths of terminal branches for congeners are often the same or very similar. Guilds were defined by a combination of diet and feeding strategy (Kalko, 1997; Scheiner et al., 2017).

A total of 92 bat species occurred along the entire elevational gradient (Table 3, All Manu), with species richness decreasing from 76 species at 500 m to six species at 3,500 m (Cisneros et al., 2014). The distribution of species ranges along that gradient was primarily nested (table S3 of Scheiner et al., 2017), exhibiting clumped species‐loss with increasing elevation (Patterson et al., 1998). Spatially restricted species–those present in only one or two sites–were found almost exclusively at low elevations, although a few species were found only at mid‐ or high elevations.

**Table 3:** Phylogenetic diversity metrics for bats from Manu Biosphere Reserve

Dataset	*S*	*M*(*P*)	1 *E*(*P*)	*M*(*PR*)	1 *D*(*P*)
All Manu	92	0.26	0.87	24.32	79.88
Elevational transects					
500 m asl	76	0.26	0.46	19.57	34.94
750 m asl	64	0.24	0.26	15.26	16.71
1,000 m asl	52	0.22	0.16	11.22	8.36
1,250 m asl	45	0.19	0.12	8.61	5.40
1,500 m asl	26	0.18	0.10	4.59	2.52
1,750 m asl	19	0.17	0.10	3.26	1.94
2,000 m asl	15	0.18	0.12	2.75	1.73
2,250 m asl	11	0.19	0.14	2.12	1.52
2,500 m asl	14	0.24	0.13	3.29	1.84
2,750 m asl	11	0.24	0.15	2.66	1.63
3,000 m asl	8	0.19	0.17	1.53	1.35
3,250 m asl	7	0.18	0.18	1.28	1.29
3,500 m asl	6	0.17	0.21	1.03	1.23
α‐diversity	27.23	0.20	0.18	5.94	3.08
β‐diversity	3.38	1.30	4.92	4.10	25.97
Guilds					
Aerial insectivores	22	0.39	0.19	8.56	4.16
Canopy frugivores	17	0.14	0.10	2.43	1.67
Gleaning omnivores	16	0.26	0.13	4.10	2.14
High‐flying insectivores	3	0.67	0.45	2.01	1.35
Understory frugivores	17	0.16	0.10	2.21	1.76
Nectarivores	11	0.20	0.14	0.38	1.55
Piscivores	1	0.38	1.07	0.85	1.07
Sanguinivores	2	0.42	0.58	2.78	1.15
α‐diversity	11.13	0.33	0.34	2.92	1.69
β‐diversity	8.27	0.80	2.52	8.34	47.20

*S*: species richness; *M*(*P*): Mean proportional branch length; ^1^ *E*(*P*): Phylogenetic evenness; *M*(*PR*): Chao's phylogenetic richness; ^1^ *D*(*P*): Phylogenetic diversity.

Divergence [*M*(*P*)] was greatest in lowland rainforest and at the transition between cloud and elfin forests (2,500 and 2,750 m); it was least in upper elfin forests (>3,000 m) and in cloud forests (1,500–2,250 m). Regularity [^1^ *E*(*P*)] also was greatest in lowland rainforest, but dipped to its smallest values in mid‐elevation cloud forests and increased in high elevation elfin forests. However, even at its largest values, regularity for a particular community was substantially lower than that of the total metacommunity (i.e., data for all strata). Combined with the general nestedness of the species along the elevational gradient, these patterns indicate that the first species to drop out with increasing elevation are closely related to other species in the metacommunity, typically members of species‐rich clades that are dominated by frugivores or gleaning omnivores (Figure 1). As a result, measures of the effective number of phylogenetically independent or distinct species [*M*(*PR*) and ^1^ *D*(*P*)] decreased very quickly with elevation, and β‐diversity values were high.

This pattern contrasts with that of functional biodiversity, where functional evenness was high along the entire elevational gradient, the effective number of functionally distinct species was much closer to species richness, and β‐diversity among elevational strata was low (Scheiner et al., 2017). If functional trait values provide a measure of the potential for resource competition among species, then there must be other, or more subtle, differences among closely related species that cause them to be preferentially excluded with increasing elevation. Importantly, these contrasting patterns only become apparent because we can compare phylogenetic and functional patterns in units of the effective number of species.

A comparison of guilds (Table 3, Figure 1) reveals a different effect of phylogenetic patterning. Unlike elevationally defined communities, each guild contains a unique set of species. Focusing on the five guilds at Manu with more than 10 species (i.e., aerial insectivores, canopy frugivores, understory frugivores, gleaning omnivores, and nectarivores), both measures of the effective number of species [*M*(*PR*) and ^1^ *D*(*P*)] are much lower than species richness. This difference indicates that each of the guilds generally includes several distantly related clades such as vespertilionids, thyropterids, and emballonurids for the aerial insectivores, or members of the Lonchorhinini, Micronycterini, and Vampyrini tribes for the gleaning omnivores. Along the elevational gradient (Figure 1), each guild generally loses some species from each clade–rather than loosing entire clades–explaining the maintenance of functional diversity as phylogenetic diversity decreases along the elevational gradient.

Phylogenetic diversity is based on evolutionary relationships in one dimension, time. In contrast, function diversity can be based on multiple aspects of species function or multiple niche axes. A detailed consideration of relationships between phylogenetic divergence and functional dispersion found that dispersion of foraging strategy and wing aerodynamics were each positively related to phylogenetic divergence, whereas diet, foraging location, masticatory mode, and body size were not (Cisneros et al., 2014). Importantly, all of the functional traits were related to resource use and acquisition; none were related to others factors that can determine species distributions along the environmental gradient such as physiological tolerances. Changes in functional diversity of bats along the elevational gradient at Manu are driven by decreasing diversity and abundance of resources with increasing elevation, resulting in both the loss of entire guilds and a reduction of functional redundancy within guilds (Cisneros et al., 2014; Scheiner et al., 2017). Studies of niche lability show that aspects of habitat or climatic niche may be more labile than others (e.g., Broennimann et al., 2007; Kozak & Wiens, 2010). It is easier to adapt to perform the same function in a different habitat or under different physiological conditions than it is to adapt a different diet or foraging strategy, as appears to be the case for bats at Manu. Most species and some guilds are restricted to low elevations; however, members of many guilds (aerial insectivores, high‐flying insectivores, canopy frugivores, understory frugivores, nectarivores, gleaning animalivores) and many clades have adapted to perform similar functions to those of close relatives but do so in the different forest types and climatic conditions of higher elevations (Figure 1). The labile nature of these niche characteristics may explain the different patterns of functional and phylogenetic diversity along this extensive environmental gradient.

## 5 DISCUSSION

### 5.1 The value of a comprehensive framework

The creation of a comprehensive framework for biodiversity metrics (Table 1) organizes and clarifies the growing panoply of ways to translate different concepts of biodiversity into quantitative indices. Our framework is grounded in two core characteristics–magnitude and variability–that are shared by different types of information–abundance, phylogeny, and function. This framework allows meaningful comparisons among metrics based on each type of information, alone or in combination. For example, the framework clarifies the properties measured by three metrics [^*q*^ *D*_*I*_(*AP*), ^*q*^ *D*(*AP*), and ^*q*^ *D*(*P*)^*T*^] that combine abundance and phylogenetic information using a Hill approach. As important, the framework provides insights into ecological and evolutionary processes that structure communities. Tucker et al. (2017, figure 5) illustrate such insights by listing a series of questions that pertain to different types of phylogenetic diversity: divergence, regularity, and phylogenetic richness. Consider one question: Is environmental filtering more important in high‐elevation communities than in low‐elevation communities? In comparisons with bat communities along an elevational gradient, we found that regularity (phylogenetic magnitude) was higher (i.e., less filtering) at low elevations (Table 2). But this was true only for phylogenetic relationships. Dispersion (functional magnitude) showed no elevational relationship (Scheiner et al., 2017). This comparison among types of information is possible because our framework makes clear which phylogenetic and functional diversity metrics are measuring analogous properties using a similar mathematical approach.

The questions posed by Tucker et al. (2017) are only an illustrative subset of the many types of questions that can be addressed within a comprehensive biodiversity metric framework, and we will not replicate their extensive discussion of this issue. Magurran and McGill (2011) provide a broad discussion covering all of the types of information that may be used to measure biodiversity. Missing from that discussion is an explicit comparison of phylogenetic and functional metrics, although such a comparison is implicit in one of the questions posed by Tucker et al. (What is the relationship between evolutionary history and variation in function?). When only one type of information is available (usually phylogeny) it is often used as a proxy for the other type based on an assumption of niche conservatism (e.g., Connolly et al., 2011; Gerhold, Cahill, Winter, Bartish, & Prinzing, 2015). However, as illustrated by the bats of Manu, and as has been shown elsewhere (e.g., Kluge & Kessler, 2011; Narwani, Alexandrou, Oakley, Carroll, & Cardinale, 2013; Purschke et al., 2013), one type of information does not necessarily reflect the other. Making effective comparisons requires that the same properties of each type of information are reflected in the metrics. A comparison of regularity information (phylogenetic variability) with dispersion information (functional magnitude) for bats at Manu would show similar patterns along the elevational gradient. But such a comparison is based on different properties for the concepts that are being compared, and may not answer a question of ecological or evolutionary importance or interest. The selection of metrics for comparative analyzes requires careful consideration of the question(s) being posed and process(es) being tested (Tucker et al., 2017).

Application of clearly defined metrics also facilitates comparisons among taxa. For bats at Manu, low elevations harbor many clades and high elevation support few, closely related clades as indicated by declining phylogenetic diversity. In a study of hummingbirds across the Andes, Graham, Parra, Rahbek, and McGuire (2009) found a similar pattern. The use of the same phylogenetic diversity metrics for the two groups would more precisely facilitate a direct comparison, possibly revealing finer scale similarities or differences.

### 5.2 Our new metrics

In this study, we present new metrics of phylogenetic diversity that reflect concepts not included in other indices, measures of β‐diversity for regularity and a new concept, phylogenetically weighted effective mean abundance. These expansions provide additional tools for addressing questions about the ecological and evolutionary processes that structure patterns of biodiversity.

We did not consider statistical issues involved in the application of our new metrics. For example, estimates of Faith's PD are highly dependent on sample size and completeness. These dependencies can be addressed using analytical approaches for rarefaction and extrapolation or bootstrap techniques for confidence estimation. Hsieh and Chao (2017) demonstrate how to do so for the phylogenetic metrics of Chao et al. (2010), expanding on previous efforts for abundance‐based Hill numbers (Chao et al., 2013). Such approaches would likely be fruitful for our metrics.

Understanding phylogenetic relationships is highly dependent on the pattern and completeness of taxon sampling: the extent to which the sampled species span the entire cladogram of a group (Nabhan & Sarkar, 2012). For phylogenetic diversity metrics, incompleteness may come from two causes: a failure to observe rare taxa, or the absence of some taxa within the domain of interest (e.g., the Andes). A failure of observation can be addressed with the techniques of Hsieh and Chao (2017). The absence of taxa is a different matter. For example, of the three extant three sanguinivorous bats, two are at Manu data (Table 3). The third is absent, not because of a sampling effect, but because it does not actually occur in the region. The lack of such a taxon can affect data interpretations. Environmental filtering is predicted to cause lower divergence in local communities compared to the regional species pool (Cavender‐Bares & Wilczek, 2003). The filtering, however, may have occurred at a continental scale, rather than within the local region. For some groups (e.g., amniotes, vascular plants), taxon sampling and phylogeny construction are relatively complete on a worldwide basis so that comparisons of phylogenetic diversity metrics based on regional, continental, or global species sets might be warranted.

### 5.3 Next steps

Numerical simulations provide useful information for selecting an appropriate index from among the myriad of available metrics of biodiversity (e.g., Mouchet, Villéger, Mason, & Mouillot, 2010; Tucker et al., 2017) or empirical data (e.g., Pavoine, 2016; Scheiner et al., 2017). For example, Tucker et al. showed that two metrics of regularity that were measured in different ways–pairwise distance (VPD) and phylogenetic isolation (var(ED))–resulted in similar estimates of phylogenetic diversity. Such analyzes identify which metrics respond in similar ways to changes in tree topology, branch length, or other cladogram properties, and which respond in different ways and so provide complementary information. Such comparisons need to be accompanied with an analysis of their mathematical bases that reaches across information types, as was done by Chiu et al. (2014) for phylogenetic and abundance information.

Our proposed framework (Table 1) can help select a metric based on the ecological or evolutionary question(s) of concern, the correspondence between the properties of possible metrics and the concepts of interest defined by the questions, and an understanding of how particular metrics respond to variation in the parameters that compose them. A revisiting of table 1 of Tucker et al. (2017) is warranted in light of our conceptual framework, for example, identifying which metrics are composites of phylogenetic and identity or abundance information. A similar effort is needed for functional diversity metrics.

The goal of our efforts is to provide a set of tools–biodiversity metrics–that can be used to study ecological and evolutionary processes and to enable setting conservation priorities. Systematizing the myriad metrics that have proliferated during the past decade will make that task easier by revealing mathematical properties of metrics, the relationships among metrics, and perhaps most importantly, the extent to which ecological or evolutionary concepts are incarnate in particular metrics. Our framework is a step in that direction.

## CONFLICT OF INTEREST

None declared.

## AUTHOR CONTRIBUTIONS

All authors contributed to the development of the ideas, metrics, and the writing of the manuscript. The data were compiled by SP and MW, and analyzed by SP and SS.
